# Complete resection of a duodenal submucosal tumor using clip-and-snare assisted endoscopic submucosal resection: a case report

**DOI:** 10.1055/a-2781-5978

**Published:** 2026-02-09

**Authors:** Can Wu, Mingliang Feng

**Affiliations:** 1 159407 Department of Gastrointestinal Endoscopy, The First Hospital of China Medical University, Shenyang, China

The histopathological types of gastrointestinal submucosal tumors (SMTs) are complex, but most are benign lesions. For small superficial SMTs with intraluminal growth (located in the mucosa or submucosa), endoscopic mucosal resection or endoscopic submucosal dissection (ESD) can be employed for removal. Due to the unique anatomical structure of the duodenum, performing endoscopic resection for duodenal SMTs requires advanced technical skills and carries a higher risk of intraoperative complications 1 2 . Recent studies have shown that, for small superficial SMTs in the rectum, the modified clip-and-snare assisted endoscopic submucosal resection (CS-ESMR) technique can effectively reduce intraoperative bleeding and operation time and lower hospitalization costs, while improving the R0 resection rate 3 . Recently, we successfully and safely resected a duodenal SMT using the CS-ESMR technique, and postoperative pathology confirmed it as a leiomyoma.

**Fig. 1:**
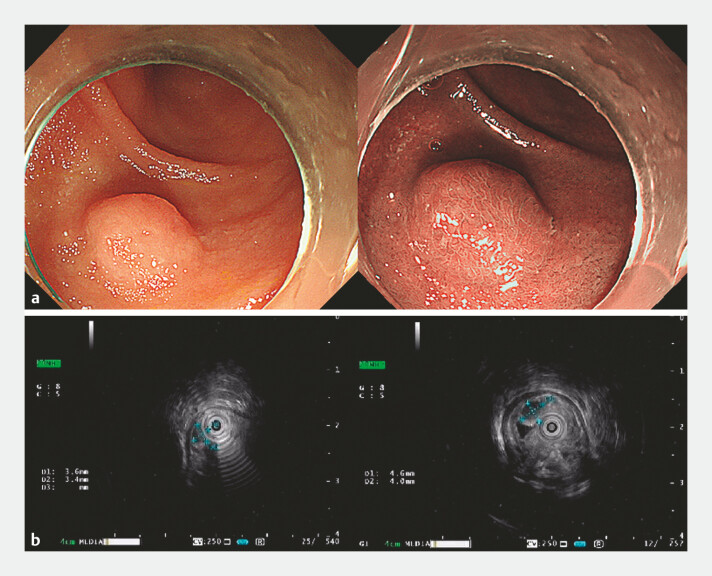
A submucosal protrusion was observed in the descending part of the duodenum, with a smooth surface. **a** In NBI mode, the surface glands appeared villous and arranged in a regular pattern. **b** Endoscopic ultrasonography revealed a lesion measuring approximately 4.6 × 4.0 mm, protruding into the lumen, and originating from the submucosal layer. NBI, narrow band imaging.

**Fig. 2:**
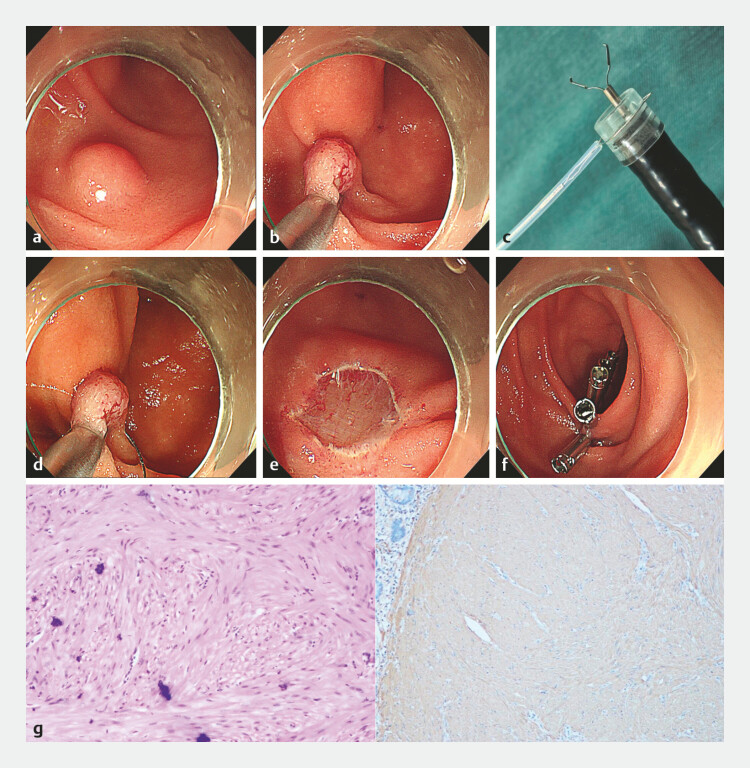
Endoscopic resection of a duodenal submucosal tumor using the clip-and-snare assisted endoscopic submucosal resection (CS-ESMR) technique. **a** The lesion was located on the posterior wall distal to the major duodenal papilla in the descending duodenum. **b** The metal clip clamped the base of the protruding lesion and completely lift it. **c** The snare was pre-positioned outside the single-channel endoscope with a transparent cap, and the metal clip was delivered through the endoscope working channel. **d** The pre-installed snare fully encircled the base of the lesion and completely resect it. **e** The wound was intact, with no active bleeding and no muscular layer injury. **f** The resection site was completely closed using metal clips. **g** Histological appearance confirming the resected specimen as a leiomyoma with negative margins.

A 65-year-old female patient underwent endoscopic treatment for a submucosal protrusion in the descending segment of the duodenum ( Fig. 1 **a** ). Endoscopic ultrasonography revealed a lesion measuring approximately 4.6 × 4.0 mm, protruding into the lumen, and originating from the submucosal layer ( Fig. 1 **b** ). Therefore, it was considered to use CS-ESMR for complete resection ( Video 1 ). The lesion was located on the posterior wall distal to the major duodenal papilla in the descending duodenum ( Fig. 2 **a** ). We first attempted to capture the tumor using a metal clip, and the tumor was successfully lifted, which was consistent with the endoscopic ultrasonographic finding. Then, we placed the snare outside the single-channel endoscope with a transparent cap and used the endoscope to send the snare to the lesion location. Through the endoscope working channel, we clamped the metal clip at the base of the protruding lesion, completely lifting it ( Fig. 2 **b** ). Then, guided by the metal clip, we used the pre-installed snare to fully encircle the base of the lesion ( Fig. 2 **c** ) and completely resect it ( Fig. 2 **d** ). The wound was intact, with no active bleeding and no muscular layer injury ( Fig. 2 **e** ). Finally, the resection site was completely closed using metal clips ( Fig. 2 **f** ). The patient recovered well postoperatively without adverse reactions such as fever or abdominal pain. The postoperative pathological examination showed that it was a leiomyoma with negative margins ( Fig. 2 **g** ).

Clip-and-snare assisted endoscopic submucosal resection of a duodenal submucosal tumor. Using a metal clip to grasp the base of the submucosal lesion, elevating it completely, allowing a pre-loaded snare to surround and resect it endoscopically. The surgical wound is closed with clips.Video 1

Compared with other parts of the gastrointestinal tract, endoscopic treatment of duodenal lesions is more challenging, prone to complications, and the complications are often more dangerous and difficult to manage 1 . CS-ESMR is a simpler and faster procedure with a significantly lower complication rate than ESD. And, it has been successfully applied in the endoscopic resection of an esophageal SMT lesion in the recent case 4 . However, the available data primarily come from studies on rectal lesions 3 . The duodenal mucosa is softer and more flexible. Therefore, for smaller lesions confined to the submucosal layer of the duodenum, CS-ESMR is simpler and safer than traditional endoscopic resection techniques and is expected to become a novel minimally invasive endoscopic treatment method that is easy to apply and promote in clinical practice.

Endoscopy_UCTN_Code_CPL_1AH_2AZ_3AC
